# Hyperimmune plasma in three immuno-deficient patients affected by non-severe, prolonged COVID-19: a single-center experience

**DOI:** 10.1186/s12879-021-06321-2

**Published:** 2021-07-01

**Authors:** Maria Grazia Cusi, Edoardo Conticini, Claudia Gandolfo, Gabriele Anichini, Gianni Gori Savellini, Serafina Valente, Federico Franchi, Sabino Scolletta, Elena Percivalle, Bruno Frediani

**Affiliations:** 1 Virology Unit, Department of Medical Biotechnologies, University of Siena, Siena, Italy; 2 Rheumatology Unit, Department of Medicine, Surgery and Neurosciences, University of Siena, viale Mario Bracci, 16, Siena, Italy; 3 Clinical and Surgical Cardiology Unit, Cardio-Thoracic and Vascular Department, University Hospital of Siena, Siena, Italy; 4 COVID Unit, University Hospital of Siena, Siena, Italy; 5 Anesthesia and Intensive Care Unit, Department of Medicine, Surgery and Neuroscience, University of Siena, Siena, Tuscany Italy; 6 Microbiology /Virology Unit, Department of Diagnostic Medicine, Fondazione IRCCS San Matteo, Pavia, Italy

**Keywords:** Convalescent plasma, Hyperimmune plasma, SARS-CoV2, COVID-19, Immunodeficiency

## Abstract

**Background:**

Convalescent plasma (CP) and hyperimmune plasma (HP) are passive immunotherapies consisting in the infusion of plasma from recovered people into infected patients. Following pre-existing evidence in many other viral diseases, such as SARS, MERS and Ebola, CP and HP have also been proposed for the treatment of COVID-19. Nevertheless, due to the lack of large, well-designed, clinical trials, no clear-cut guidelines exist about what subtype of patient CP and HP should be administered to.

**Case presentation:**

We have reported the cases of 3 patients, all immunosuppressed and affected by non-severe, prolonged COVID-19. They were treated with HP, whose neutralizing titer was higher than 1/80. The first patient was a 55-year-old male, who had undergone lung transplant. He was under therapy with Tacrolimus and developed non-neutralizing antibodies against SARS-CoV2. The second patient was a 77-year-old female, affected by follicular lymphoma. She had tested positive for SARS-CoV2 after 6 months. The third was a 60-year-old patient, affected by chronic leukemia. He did not develop antibodies after 2-month disease. All 3 patients received HP and had tested negative for SARS-CoV2 within 2 weeks.

**Conclusion:**

Despite encouraging initial data, no strong evidence exist in support of CP and HP to treat COVID-19. In our experience, although limited due to the reduced number of patients, we found a good safety and efficacy of HP in 3 immuno-deficient subjects. Further data are needed in order to assess whether this subtype of patients may particularly benefit from passive immunization.

## Background

Convalescent plasma (CP) is a promising therapy to treat patients affected by Coronavirus disease 2019 (COVID-19) [1, 2]. CP is a passive immunotherapy consisting in the infusion of plasma from recovered people into infected patients and it is thought to act by transferring neutralizing antibodies [3]. Hyperimmune plasma (HP) is another blood component sharing the same mechanism of action of CP, but with the advantage of a better standardization in terms of neutralizing antibodies titer, lower volume, and easier storage, no need of group matching, these *pros* are nevertheless counterbalanced by a lower availability [4].

Despite some encouraging data coming from the first published papers, displaying a reduced mortality in patients treated with CP [5]), it should be remarked that only 10 of them are randomized control trials (RCTs), [5] while many others do not have a control group [6, 7]. Thus, findings from larger and well-designed clinical trials with both CP and HP are urgently needed in order to better assess efficacy and safety in COVID-19.

Moreover, an even larger uncertainty exists about the role of CP and HP in the treatment of COVID-19 patients: no clear-cut findings display whether this procedure should be performed in severely ill or asymptomatic subjects, in association with steroids, antivirals or immunosuppressive drugs, before or after the failure of a first-line treatment.

We experienced the administration of HP in 3 immunosuppressed patients, with mild to moderate disease and a prolonged positivity of nasopharyngeal swab, at the COVID Unit, University Hospital of Siena.

## Cases presentation

### Plasma collection

Collected plasma, kindly provided by the Service of Immunohematology and Transfusion Medicine, ‘San Matteo Hospital’ Pavia, Italy, showed a neutralizing titer of 1/80 or more. Donor plasma, obtained by symptomatic subjects, was tested for specific neutralizing antibody titer as previously described [8].

### Neutralizing antibody assay

Serum samples were titrated in a four-fold dilution series starting from 1/8 in 96-well tissue culture microtiter plate and mixed with a working dilution of a SARS-CoV-2 (SARS-CoV-2/human/ITA/Siena-1/2020; GenBank: 96 MT531537.2) (100TCID_50_). After 1 h incubation at 37 °C and 5% CO_2_, VERO E6 (ATCC® CRL-1586 M) cells were added. After 72 h incubation, the cultures were daily examined under the microscope (Olympus 120 IX51) for the presence of the cytopathic effect (CPE). The 50% end point titer was calculated using the Reed-Muench method [9]. A positive and negative control serum was included in each assay. A positive titer was equal to or greater than 1/20.

### Molecular testing

Nasopharyngeal swabs were analyzed by using the Allplex 2019-nCoV assay (Arrow Diagnostics S.r.l., Italy) for molecular testing. The analysis included genes encoding the envelope (E), the RNA-dependent RNA polymerase (RdRp) and the nucleocapsid (N). A cycle threshold (*C*_*T*_) value of less than 40 was defined as a positive test, while a *C*_*T*_ value of 40 or more was considered as a negative test.

### Patient 1

The first patient treated with HP was a 55-year-old male, who had undergone lung transplant due to a congenital bullous emphysema and was under therapy with oral glucocorticoids and Tacrolimus. He had previously suffered from diffuse alveolar damage, leading to lung fibrosis and Aspergillus fumigatus infection in the transplanted lung. He had tested positive for SARS-CoV2 in April 2020, despite the presence of IgG, which were found to be non-neutralizing (titer< 1/20). Further tests after two months evidenced a persistent positivity of nasopharyngeal swab (E Ct 17.5, RdRp Ct 18.3 and N Ct 18.2). Lymphocytes subpopulation evidenced very low levels of B cells (28/ul, normal value 90–660). In June, he was treated with two administrations of HP compatible for ABO and RhD grouping and neutralizing titer of 1/80. Seven days since the second infusion, he had eventually tested negative for SARS-CoV-2.

### Patient 2

The second patient, a 77-year-old female suffering from mild symptoms (ageusia, anosmia, fever) had tested positive in April. Her previous medical history evidenced gastric follicular lymphoma, in treatment with Rituximab and Bendamustine, and ulcerative colitis, for which the patient was assuming Mesalazine. After 6 months, further nasopharyngeal swabs displayed a persistent positivity (E Ct 31.6, RdRp Ct 34 and N Ct 32.2) and no specific IgG or IgM antibodies were developed. Total serum IgG and IgM were below normal values, too. For these reasons, the patient underwent two administrations of HP ABO and RhD grouping and neutralizing titer of 1/80. She was discharged with low viral load and eventually tested negative 7 days later.

### Patient 3

The third and last patient of our case series was a 60-year-old male, affected by stage IV chronic lymphocytic leukemia and in treatment with Bendamustine, Cotrimoxazole and Aciclovir. He had been admitted to our COVID Unit with a diagnosis of bilateral interstitial pneumonia and blood examination revealed leukopenia and thrombocytopenia.

After two months, the patient did not develop any specific antibody response against SARS-CoV-2 and his nasopharyngeal swab was persistently positive (E Ct 24.3, RdRp Ct 24.3 and N Ct 23), thus he was treated with two administrations of HP ABO and RhD grouping and neutralizing titer of 1/100. The patient was subsequently discharged and he had eventually tested negative after two weeks.

## Discussion and conclusions

Due to the lack of effective drugs in the treatment of COVID-19, several authors [1–3] have proposed the use of CP and HP. Passive immunization proved variable efficacy in several infectious diseases, such as Spanish flu, Middle East Respiratory Syndrome (MERS), Severe Acute Respiratory Syndrome (SARS) and Ebola [7, 8, 10–12], all linked by a similar physiopathology of the lung damage.

To date, more than 100 trials have been registered worldwide, but only few data have been published [5, 6], providing conflicting data in terms of mortality, duration and progression of disease in hospitalized, critically ill, subjects treated with CP [5, 6, 13]. Moreover, all these studies, as well as the other ones stopped or still ongoing, are affected by a notable variety of the sample and by the uncertainty of the serological status of the patients, since many of them had anti-SARS-CoV-2 antibodies.

HP improved radiological findings and reduced mortality, markers of inflammation and viral load in an Italian cohort of moderate-to-severe COVID-19 patients [8], but, due to the lack of a control group, these findings still have to be confirmed in larger controlled studies. Indeed, no RCT investigating the efficacy of HP in COVID-19 has been published yet [6].

On the other hand, CP and HP appear to be quite safe, with no serious or unexpected adverse events (AEs) in the majority of patients who underwent this treatment [14, 15]. Nevertheless, no clear-cut-conclusion can be drawn about the safety of CP and HP, due to the limited information about grade 3 and 4 adverse events (AEs) [5].

Moreover, as previously mentioned, a profound uncertainty exists about what subset of COVID-19 patients may benefit from passive immunization and when the treatment is more efficacious during the course of the disease. Data from the currently available literature seem to suggest that, in case of severe COVID-19 infection, the highest efficacy of the treatment is achieved when CP is infused within the first 7 days [16].

Conversely, in our experience, we chose to administer HP in 3 patients affected by different conditions (hematological malignancies and organ transplant) leading to prolonged immunodeficiency, with a marked reduction in number and functionality of B lymphocytes. All these patients had been previously treated with intravenous and/or oral steroids and, due to their comorbidities, they were not considered eligible for further pharmacological treatments.

HP was effective in reducing viral load in all patients and led to their hospital discharge, with no further complications, AEs or need to invasive ventilation.

It is well known that the major complication of severe COVID-19 infection is an acute respiratory distress syndrome (ARDS), presumably mediated by an aberrant and exaggerate response of immune system. On the other hand, immunodeficient patients probably lack those immune stimuli leading to ARDS [17] but, conversely, they also lack defense mechanisms involved in viral clearance and antibodies production and associated to a higher viral load and a lower viral clearance [18, 19].

For these reasons, if a combined treatment of both antiviral and immunosuppressive drugs may represent a promising option for the first and more common subtype of COVID-19 patients, CP or HP should be administered to all those subjects whose immune system is impaired by concomitant treatments or diseases. Indeed, passive immunization, whose antiviral activity may probably not provide substantial benefits in severely ill patients, may be conversely more indicated in those subjects at risk for a sudden worsening, due to an impaired viral clearance [4].

A growing number of evidence is currently witnessing the potential role of passive immunization in patients affected by different conditions, such as malignancies, congenital and acquired immunodeficiencies and organ transplants, all with an impaired immune humoral response [20].. Nevertheless, these data only rely on case reports or case series, and no RCT has currently included immunocompromised subjects. At the same time, the vast majority of papers focuses on CP, while only a few of them have reported the use of HP. [21]).

In conclusion, our findings, although limited by the small number of patients, provide some interesting insights: first, HP also proved to be effective and safe in fragile and compromised subjects, burdened by severe comorbidities, low life expectancy and prolonged duration of disease; secondly, our paper may help paving the way in defining a tailored therapy for particular subsets of patients.

Due to the limited number of patients, we cannot exclude the risk of re-infection in these patients connected with their persistent immunosuppression; however, the HP treatment was successful to make them COVID-free. The patients were followed-up after discharge and they did not show persistence of the virus three months later. Nevertheless, the recent emergence of variants is underscoring the need to use HP more wisely, particularly taking into account the epidemiological picture and considering the circulating viral variants in specific geographic areas. This aspect is aiming to use only HP having antibodies which can cross-neutralize the viral variant.

## Data Availability

The datasets generated during and/or analysed during the current study are available from the corresponding author on reasonable request.

## Abbreviations

AEs: Adverse events
ARDS: Acute respiratory distress syndrome
COVID-19: Coronavirus disease-19
CP: Convalescent plasma
HP: Hyperimmune plasma
MERS: Middle East Respiratory Syndrome
SARS: Severe Acute Respiratory Syndrome
